# Developing an Efficient and General Strategy for Immobilization of Small Molecules onto Microarrays Using Isocyanate Chemistry

**DOI:** 10.3390/s16030378

**Published:** 2016-03-16

**Authors:** Chenggang Zhu, Xiangdong Zhu, James P. Landry, Zhaomeng Cui, Quanfu Li, Yongjun Dang, Lan Mi, Fengyun Zheng, Yiyan Fei

**Affiliations:** 1 Department of Optical Science and Engineering, Shanghai Engineering Research Center of Ultra-Precision Optical Manufacturing, Key Laboratory of Micro and Nano Photonic Structures (Ministry of Education), Fudan University, Shanghai 200433, China; 13210720017@fudan.edu.cn (C.Z.); lanmi@fudan.edu.cn (L.M.); 2 Department of Physics, University of California, Davis, CA 95616, USA; xdzhu@physics.ucdavis.edu (X.Z.); jim.landry@gmail.com (J.P.L.); 3 Key Laboratory of Metabolism and Molecular Medicine, the Ministry of Education, Department of Biochemistry and Molecular Biology, School of Basic Medical Sciences, Fudan University, Shanghai 200032, China; 15111010017@fudan.edu.cn (Z.C.); lqfmic@gmail.com (Q.L.); yongjundang@fudan.edu.cn (Y.D.); 4 Institutes of Biomedical Sciences, Fudan University, Shanghai 200032, China; zhengfengyun@outlook.com

**Keywords:** high-throughput screening, small-molecule microarrays, surface functionalization, label-free optical biosensor

## Abstract

Small-molecule microarray (SMM) is an effective platform for identifying lead compounds from large collections of small molecules in drug discovery, and efficient immobilization of molecular compounds is a pre-requisite for the success of such a platform. On an isocyanate functionalized surface, we studied the dependence of immobilization efficiency on chemical residues on molecular compounds, terminal residues on isocyanate functionalized surface, lengths of spacer molecules, and post-printing treatment conditions, and we identified a set of optimized conditions that enable us to immobilize small molecules with significantly improved efficiencies, particularly for those molecules with carboxylic acid residues that are known to have low isocyanate reactivity. We fabricated microarrays of 3375 bioactive compounds on isocyanate functionalized glass slides under these optimized conditions and confirmed that immobilization percentage is over 73%.

## 1. Introduction

Using large arrays of small molecules immobilized on a solid support (small-molecule microarrays or SMMs), the reaction of a protein probe with thousands of distinct molecules can be characterized simultaneously in a single experiment. As a result, SMMs have emerged as one of the high-throughput screening platforms for probing of protein-ligand interactions [1] in applications such as drug discovery [2,3], protein profiling [4], ligand discovery [5], and biomarker search [3,6].

The success of microarray-based screening platforms hinges on efficient immobilization of a wide variety of small molecules on solid supports. A number of immobilization schemes exist for SMM fabrication, based on selective or non-selective attachment methods. Examples of selective attachment schemes include immobilization of biotin groups on strepavidin functionalized surface [7,8,9,10,11,12,13], thiol groups on a maleimide functionalized surface [14], alcohol groups on a silyl chloride functionalized surface [15], phenol or carboxylic acid groups on a diazobenzylidene functionalized surface [16], hydrazide groups on an epoxide functionalized surface [17], azide groups on an alkyne [18] or phosphane functionalized surface [19], polyfluorocarbon groups on a fluoroalkylsilane functionalized surface [20] and peptide-nucleic acids on an oligonucleotide functionalized surface [21]. These selective attachment methods ensure that the surface-immobilized biomolecules retain most of their original activities uniformly. However, they are not applicable to libraries of natural products or other compound libraries without a common residue for anchoring. In these cases, non-selective attachment methods such as photo-activated cross-linking and capture of nucleophilic residues by isocyanate-functionalized surface are alternatives. The photo-activated cross-linking method provides universal immobilization via carbon-based random insertion, but it comes with relatively high false positive results [22,23]. An isocyanate-functionalized surface is covalently reactive to compounds containing nucleophilic residues and thus can be used to immobilize molecules non-selectively [24,25]. SMMs of natural products, commercially available compound libraries, and oligonucleotides have been successfully fabricated on isocyanate functionalized glass slides and used in drug discovery and genomics study [26,27,28,29,30,31,32]. 

One issue concerning the isocyanate capture method is the relatively low immobilization efficiency for some molecules due to their low reactivity with isocyanate. Among nucleophilic residues, primary amine, aryl amine, and thiol have high reactivity to isocyanate; primary alcohol, phenol, and secondary amine have medium reactivity to isocyanate; carboxylic acid, secondary alcohol, and tertiary alcohol have low reactivity to isocyanate. The immobilization efficiency of molecules with carboxylic acid and secondary alcohol is less than 10% that of molecules with primary amine [23]. In screening 8000 compounds on SMMs for ligands of vascular endothelial growth factor (VEGF) [27] Fei and coworkers identified 64 hits with nucleophilic residues that bound to VEGF. Among them, 21 (33%) compounds have high isocyanate reactivity, 36 (56%) compounds have medium isocyanate reactivity, and 7 (11%) compounds have low isocyanate reactivity. The number of hits with low isocyanate reactivity is much smaller than the number of compounds with medium or high isocyanate reactivity. It is feasible that compounds with nucleophilic residues of low isocyanate reactivity are much less immobilized than compounds with nucleophilic residues of medium or high isocyanate reactivity under the same microarray fabrication conditions and thus fewer hits out of compounds with carboxylic acid or secondary alcohol may reflect more on the immobilization efficiency on the solid support than the reactivity of these compounds with VEGF. It is thus important to find optimal ways to improve immobilization efficiencies for compounds with low isocyanate reactivity.

In this paper we report experimental studies on immobilization efficiency and morphological quality of printed small molecules on isocyanate functionalized surface as functions of (1) nucleophilic residues on small molecules for surface attachment; (2) penultimate chemical groups after terminal isocyanate residues on the isocyanate functionalized surface; (3) the length of polyethylene glycol (PEG) as the spacer between the penultimate chemical group and the glass surface; and (4) post-printing treatment. We identified a set of conditions that enable immobilization of small molecules with a wide range of nucleophilic residues, with an overall immobilization percentage of at least 73%. 

## 2. Materials and Methods

### 2.1. Reagents and Proteins

Amine functionalized glass slides were purchased from CapitalBio Corporation (Beijing, China). Polyethylene glycol (n = 1) or (PEG)_1_ was from PolyPeptide Group (San Diego, CA, USA) and (PEG)_6_ was from Shanghai Plus Bio-Science and Technology Company (Shanghai, China). (PEG)_12_, (PEG)_24_, and (PEG)_36_ were from Biomatrik Incorporated (Jiaxing, China). (PEG)_2_, N,N-Diisopropylethylamine (DIPEA), piperidine, pyridine, baicalin, hexamethylene diisocyanate (HDI), 1,4-Phenylene diisocyanate (PPDI), (benzotriazol-l-yloxy) tripyrrolidinophosphonium hexafluoro phosphate (PyBOP,) biotin-(PEG)_2_-NH_2_, biotin-(PEG)_6_-OH and D-biotin were from Aladdin Industrial Corporation (Shanghai, China). Biotin-NH-NH_2_ and biotin4-nitrophenyl ester were from Shanghai Yuanye Bio-Technology Company (Shanghai, China). N,N-Dimethylformamide (DMF), tetrahydrofuran (THF) were from Sinopharm Chemical Regent Company (Shanghai, China). Dimethyl sulfoxide (DMSO) was from Shanghai Lingfeng Chemical Reagent Company (Shanghai, China). Bovine serum albumin (BSA) was from Sigma-Aldrich (St. Louis, MO, USA) and mouse anti-biotin antibody was from Jackson ImmunoResearch Laboratories (West Grove, United states). Streptavidin (SAVD) was from Life Technologies (Shanghai, China). Biotin conjugated BSA was from Beijing Bo Sheng Bio-Technology Company Limited (Beijing, China).

### 2.2. Preparation of Isocyanate Functionalized Glass Slides

**Figure 1:**
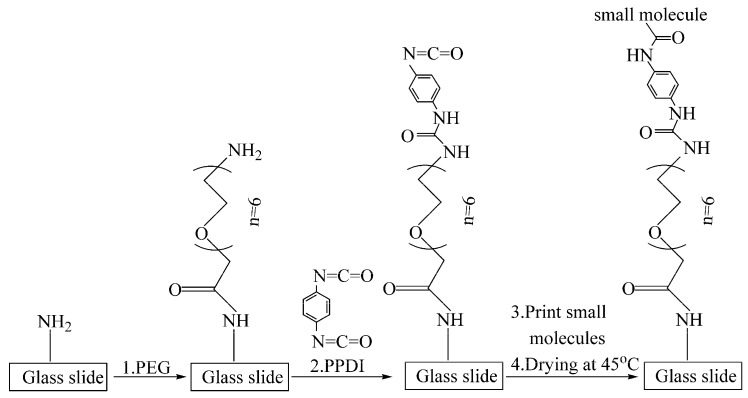
Steps of chemical modification of an amine functionalized glass slide to produce an isocyanate functionalized slide for SMM fabrication.

Figure 1 shows steps for preparing isocyanate functionalized glass slides from commercial amine functionalized glass slides. First, the spacer PEG was introduced by immersing amine functionalized glass slides into a (PEG)_n_ solution for 10 h with stirring. The (PEG)_n_ solution contained 1 mM Fmoc-NH-(PEG)_n_-(CH_2_)_2_-COOH, 2 mM PyBOP and 20 mM DIPEA dissolved in DMF. (PEG)_n_ of varying lengths (n = 1, 2, 6, 12, 24, 36) were used on respective slides. Second, the protecting Fmoc group was removed by incubating the PEG-treated glass slides in a solution of piperidine (v/v 1%) in DMF for 12 h with gentle stirring. Third, the terminal isocyanate group was added through incubation of de-protected glass slides in a solution of isocyanate for 1 hwith stirring. Two isocyanate solutions at the same concentration of 60 mM were used to add isocyanate residues with different penultimate chemical groups to the (PEG)_n_ spacer: the HDI solution in DMF was used to add hexyl-isocyanate residues and the PPDI solution in THF was used to add phenyl-isocyanate residues (Figure 1). Afterwards, isocyanate functionalized slides were rinsed with DMF and THF and dried with a stream of purified nitrogen. If not used immediately, the processed slides were stored in a −20 °C freezer until printing with small molecules.

### 2.3. Fabrication of SMMs on Isocyanate Functionalized Glass Slides

Biotinylated compound microarrays were fabricated as follows. Five biotinylated compounds, biotin-(PEG)_2_-NH_2_, biotin4-nitrophenyl ester, biotin-NH-NH_2_, biotin-(PEG)_6_-OH, and D-biotin (with intrinsic COOH group), were printed on as-prepared isocyanate functionalized glass slides with a contact microarray printer (SmartArrayer 136 Microarray Spotter, CapitalBio Corporation, Beijing, China). Each compound was diluted in DMSO and separately in a mixture of DMSO with ddH_2_O (v/v 50%) to 4 concentrations of 1 mM, 4 mM, 8 mM, and 10 mM. Three control compounds (baicalein, rapamycin, and FK506) were diluted in DMSO at a concentration of 5 mM. The biotinylated compounds were each printed in triplicate. There were a total of 135 printed spots over an area of 3.75 × 2.5 mm^2^ that formed one microarray. We printed 6 identical microarrays on one slide. The averaged diameter of printed spots was 100~150 μm and the center-to-center spot separation was 250 μm. After printing, 4 distinct post-printing treatments were used on respective glass slides to facilitate covalent attachment of nucleophilic residues to isocyanate functionalized slides: (1) drying at room temperature only; (2) drying at 45 °C only; (3) catalyzation in pyridine vapor at room temperature; and (4) catalyzation in pyridine vapor at 45 °C. After post-printing treatment, the microarrays were stored in a −20 °C freezer until use.

A small molecule microarray of 3375 bioactive compounds (FMSCL: Fudan MolMed-Selleck Compound Library), including 1053 natural compounds from Traditional Chinese Medicine (most of them from herb), 1527 drugs approved by Food and Drug Administration (FDA) and 795 known inhibitors, was prepared as follows. Each compound was dissolved in DMSO to a concentration of 10 mM and was printed in duplicate on both phenyl-isocyanate functionalized slides and hexyl-isocyanate functionalized glass slides. Biotin-BSA at a concentration of 7600 nM in 1× phosphate-buffered saline (PBS) and biotin-(PEG)_2_-NH_2_ at a concentration of 5 mM in DMSO were also printed as the inner and the outer borders of SMMs to serve as position markers and positive controls. There were 148 columns and 64 rows in each SMM over an area of 37 × 16 mm^2^. The SMM was dried at 45 °C for 24 h and were then stored in a −20 °C freezer until binding reactions with proteins.

### 2.4. Detection of SMMs with a Label-Free Ellipsometry-Based Scanning Microscope

Binding reactions of protein probes with SMMs were monitored *in situ* using an optical biosensor, scanning oblique-incidence reflectivity difference (OI-RD) microscope, whose working principle has been reported previously [33,34,35,36]. In essence, the phase change of the reflected optical beam (*i.e.*, the OI-RD signal) from the printed region is measured by the microscope and such a change is proportional to the surface mass density of protein probes that are captured by immobilized small molecules [37].

For optical detection, biotinylated compound microarrays and SMMs of 3375 bioactive compounds were assembled into respective fluidic cartridges. The microarrays were (1) washed *in situ* with a flow of 1× PBS to remove excess unbound small molecules; (2) exposed to 7600 nM BSA in 1× PBS for 30 min to block unprinted isocyanate functionalized surface; (3) exposed to anti-biotin antibody at a concentration of 86 nM for 2 h or to SAVD at a concentration of 154 nM for 1 h. The surface mass density changes in both printed and unprinted regions were determined from the optical phase difference images of the SMMs. 

## 3. Results

### 3.1. Immobilization Efficiencies of Compounds with Different Nucleophilic Residues Printed on Hexyl-Isocyanate Functionalized Slides

**Figure 2:**
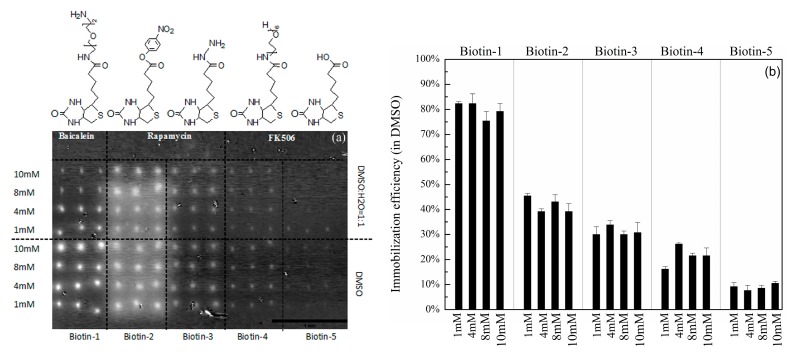
(**a**) After reaction with anti-biotin antibody, the change in OI-RD image (differential OI-RD image) of a biotinylated compound microarray on hexyl-isocyanate functionalized slide with (PEG)_6_ and catalyzed in pyridine vapor at room temperature; (**b**) Extracted immobilization efficiencies of five biotinylated compounds with different nucleophilic residues available for surface anchoring.

Hexyl-isocyanate functionalized surfaces activated with pyridine vapor at room temperature can covalently capture a variety of compounds with nucleophilic residues [25,27]. We quantified the immobilization efficiency for five biotinylated compounds on this surface, each having a distinct nucleophilic residue that reacts with the isocyanate group with different affinities. Printed microarrays of biotinylated primary amine (Biotin-1), biotinylated nitrophenyl ester (Biotin-2), biotinylated hydrazide (Biotin-3), biotinylated primary hydroxyl (Biotin-4), and D-biotin (Biotin-5) on hexyl-isocyanate functionalized slide were catalyzed with pyridine vapor at room temperature. To determine immobilization efficiencies of these compounds, the microarray was subsequently reacted with unlabeled mouse anti-biotin antibodies (with a molecular weight of 150 kDa) and the surface mass density of the antibodies captured by the immobilized compounds was measured with the OI-RD scanning microscope. Figure 2a shows that all five biotinylated compounds are indeed immobilized on the hexyl-isocyanate functionalized surface, albeit with different immobilization efficiencies as the amounts of captures mouse anti-biotin antibodies differ. From surface mass densities of the captured anti-biotin antibodies by the biotinylated compounds printed from solutions in mixtures of DMSO with ddH_2_O and by the same compounds printed from solutions in pure DMSO, we can conclude that the hexyl-isocyanate functionalized surface is not sensitive to the moisture in the printing solution.

To quantify immobilization efficiencies of compounds with different nucleophilic residues, we calculated the coverage of antibodies as the ratio of the surface mass density of antibodies captured by the compounds to that of one monolayer of antibodies in upright or “end-on” configuration. The latter is roughly 1.3 × 10^−6^ g/cm^2^, assuming that one IgG molecule measures 4.4 nm × 4.4 nm × 23.5 nm [38,39]. The coverage of captured antibodies is proportional to the amount of immobilized small molecule available to bind the antibodies. It is also proportional to the factor of [c]/([c] + K_D_), where K_D_ is the dissociation constant between protein and small molecule and [c] is the concentration of protein. Since K_D_ between unlabeled mouse anti-biotin antibody and biotin is ~1 nM [40], [c]/([c] + K_D_) is close to unity as the concentration of anti-biotin antibody [c] is 86 nM. As a result, the coverage of anti-biotin antibody can be used as the measure of the immobilization efficiency of biotinylated compounds on hexyl-isocyanate functionalized surface. Figure 2b displays the immobilization efficiencies for five printed biotinylated compounds. The immobilization efficiency for compounds with primary amine residues is about 80%; the efficiency for compounds with hydrazide and primary hydroxyl residues is about 30%; and for compounds with carboxylic acid residues, the immobilization efficiency drops to about 8%, consistent with the previous reports [23]. The immobilization efficiency is essentially unchanged when the concentration of compounds for printing varies from 1 mM to 10 mM. As expected, we found no evidence of captured mouse anti-biotin antibodies on the negative controls.

The results displayed in Figure 2 show that printing small molecules with nucleophilic residues on hexyl-isocyanate functionalized surface followed by catalyzation in pyridine vapor at room temperature produces useful small-molecule microarrays. However, immobilization efficiencies for small molecules with carboxylic acid residues and even hydrazide and primary hydroxyl residues are low, rendering subsequent binding reactions with protein probes more susceptible to background noises and drifts in label-free detection systems. 

### 3.2. Immobilization Efficiencies and Spot Morphology of Printed Compounds vs. the Length of Spacer Molecule on Hexyl-Isocyanate Functionalized Surface 

**Figure 3:**
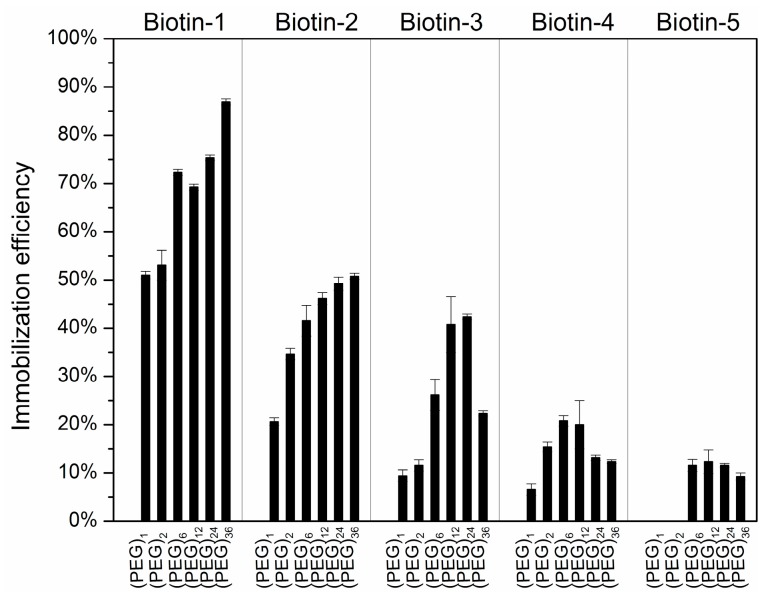
Immobilization efficiencies of biotinylated compounds on hexyl-isocyanate functionalized slide with spacers (PEG)_n_ of varying lengths (n = 1, 2, 6, 12, 24, 36).

Polyethylene glycol (PEG)_n_ is used as a spacer to separate the isocyanate residue from the solid surface so that the effect of the surface on the reactivity of subsequently immobilized compounds with protein probes in solution is reduced or minimized [41,42]. We studied the compound immobilization efficiency as a function of the length of (PEG)_n_ (n = 1, 2, 6, 12, 24, 36) (Figure 3). Clearly, the immobilization efficiencies are lessened with short PEG spacers (n = 1, 2) and we observed significant non-specific binding of protein on these surfaces. The surfaces with longer spacer (n = 6, 12, 24, 36) both improve the immobilization efficiency and reduce non-specific binding of subsequent protein probes. However, when the spacer length increases from n = 12 to n = 36, the spot morphology of printed compounds becomes more varied in terms of displacement of the spot centroid from the printed location, shape and spreading of the spot. As a result, the surface with (PEG)_6_ as the spacer gives the best immobilization result in terms of efficiency, reduction of non-specific binding, and repeatability and uniformity of spot morphology.

**Figure 4:**
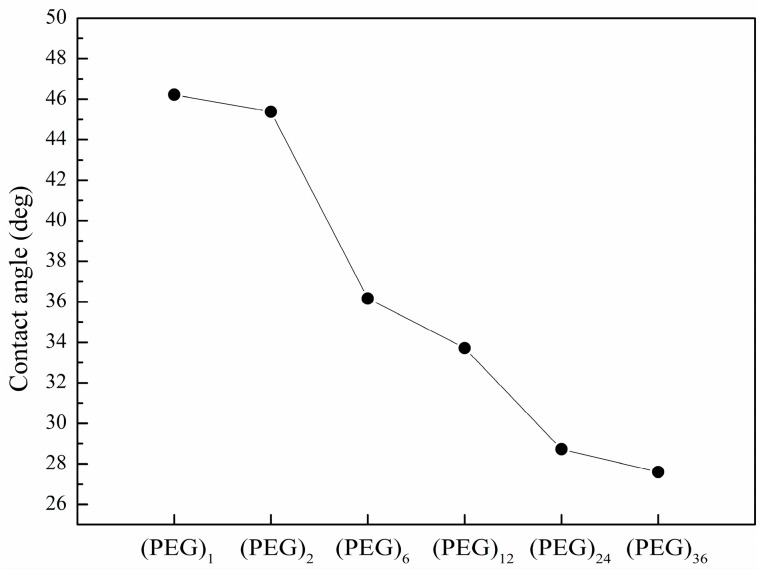
Contact angles of DMSO on hexyl-isocyanate functionalized surface with spacers (PEG)_n_ of varying lengths (n = 1, 2, 6, 12, 24, 36).

Using a contact-angle measurement system (OCA15, DataPhysics Instruments GmbH, Filderstadt, Germany), we measured the contact angles of DMSO on hexyl-isocyanate functionalized surfaces as a function of the PEG spacer length (Figure 4). The contact angle decreases as the length of the PEG spacer increases, indicating that the wettability increases with the spacer length. Printed droplets on a highly hydrophilic surface are expected to form irregular spots because of significant spreading and inhomogeneous wettability across the surface. This explains inconsistent spot morphology found on surface with long PEG spacers (n = 12, 24, 36). Consistent spot morphology is obtained with contact angle of DMSO on isocyanate functionalized surface larger than 35°.

### 3.3. Dependence of Immobilization Efficiency on the Penultimate Chemical Group to the Isocyanate Residue and Post-Printing Treatment 

**Figure 5:**
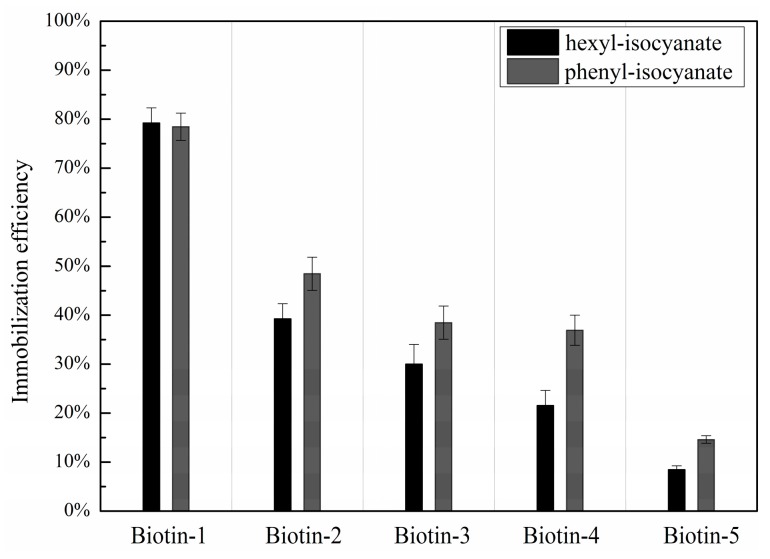
Immobilization efficiencies of five biotinylated compounds with different nucleophilic residues printed on hexyl-isocyanate surface *vs.* those printed on phenyl-isocyanate surface. The compound microarrays were both treated with pyridine vapor catalyzation at room temperature.

Figure 5 shows immobilization efficiencies of biotinylated compounds on hexyl-isocyanate functionalized and phenyl-isocyanate functionalized surfaces, after catalyzation in pyridine vapor at room temperature in both cases. The efficiencies are improved for all compounds on a phenyl-isocyanate functionalized surface when compared to a hexyl-isocyanate functionalized surface. In particular, the immobilization efficiency for compounds with carboxylic acid residues is improved by a factor of 1.7. Changing the penultimate group from hexyl to phenyl clearly enhances the attachment of nucleophiles. The presence of water in printing solutions does not affect immobilization efficiency on a phenyl-isocyanate surface, as on a hexyl-isocyanate surface.

**Figure 6:**
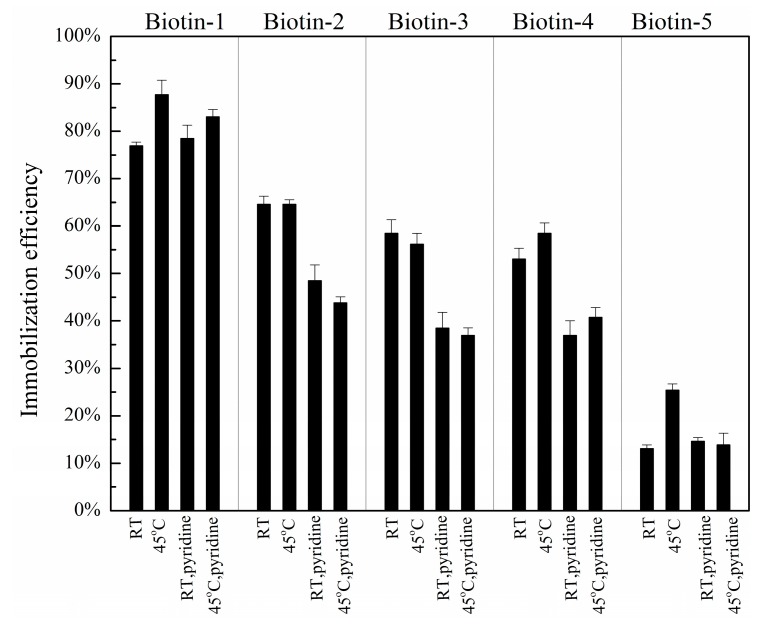
Immobilization efficiencies of five biotinylated compounds with different nucleophilic residues printed on a phenyl-isocyanate functionalized surface as functions of post-printing treatment.

We further examined the effects of post-printing treatments of compound microarrays on immobilization efficiency. In addition to catalyzation in pyridine vapor at room temperature, we explored three other treatment conditions: (1) drying printed microarrays at room temperature without catalyzation in pyridine vapor; (2) annealing printed microarrays to 45 °C without catalyzation in pyridine vapor; (3) catalyzation of printed microarrays in pyridine vapor at 45 °C (Figure 6). The immobilization efficiencies of compounds with nitrophenyl ester, hydrazide, and primary hydroxyl are improved by simply drying at room temperature instead of catalyzation in pyridine vapor at room temperature. In addition, annealing the printed microarrays at 45 °C significantly improves the immobilization efficiency for compounds with carboxylic acid residues. On the hexyl-isocyanate surface, we observed the same trend, *i.e.*, post-printing thermal annealing treatment at 45 °C alone yields the highest immobilization efficiency among all four treatment conditions.

**Figure 7:**
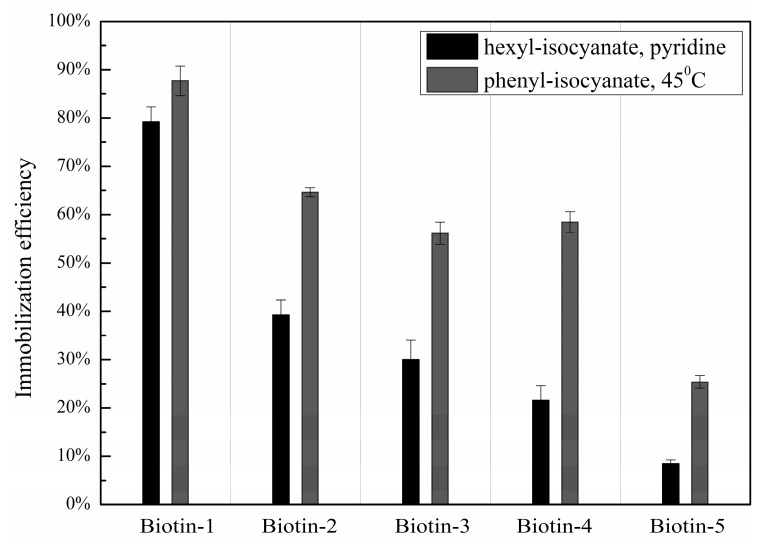
Immobilization efficiencies of five biotinylated compounds with different nucleophilic residues printed on a hexyl-isocyanate functionalized slide followed by catalyzation in pyridine vapor at room temperature *vs.* those printed on phenyl-isocyanate functionalized slide followed by thermal annealing at 45 °C for 24 h.

Compared with immobilization on a hexyl-isocyanate functionalized surface followed by catalyzation in pyridine vapor at room temperature, immobilization efficiencies of compounds are improved significantly on a phenyl-isocyanate functionalized surface followed by thermal annealing at 45 °C alone, as shown in Figure 7, to the following extents: a factor of 2 improvement for hydrazide; a factor of 3 improvement for primary hydroxyl, making it close to 60%; and a factor of 3 improvement for carboxylic acid, resulting in immobilization efficiency of 25%. Such improved immobilization efficiencies should dramatically increase the fraction of a large collection of compounds without special common handles to be efficiently immobilized on an isocyanate functionalized solid surface for screening assays. 

### 3.4. Stability of Phenyl-Isocyanate Functionalized Slides in Storage

The stability of a functionalized glass slide is important for producing reproducible binding assays as one may not use the slide immediately after fabrication. To explore this issue experimentally, we measured immobilization efficiencies of compound microarrays fabricated on phenyl-isocyanate functionalized slides that have been stored for various lengths of time before printing the compounds on the slide surface. On Day 0, we fabricated a number of phenyl-isocyanate functionalized slides. These slides were stored in a −20 °C freezer. On the day as indicated on the x axis, biotinylated compounds were printed on a slide (taken out from the freezer and thawed to room temperature) and annealed at 45 °C for 24 h. Immobilization efficiencies of the printed compounds as functions of slide storage time (Figure S1) shows that the functionality of a phenyl-isocyanate functionalized slide decreases gradually over time. For example, the efficiency for the compounds with carboxylic acid residues drops by 50% on Day 20 from the efficiency on Day 0, and disappears completely on Day 55. On a hexyl-isocyanate functionalized slide, its capability to immobilize carboxylic acids is also lost after Day 55. 

### 3.5. Immobilization of a Large Collection of Bioactive Compounds on Isocyanate Functionalized Glass Slides—Confirmation of the Benefit of the Optimal Procedures for SMM Fabrication

**Figure 8:**
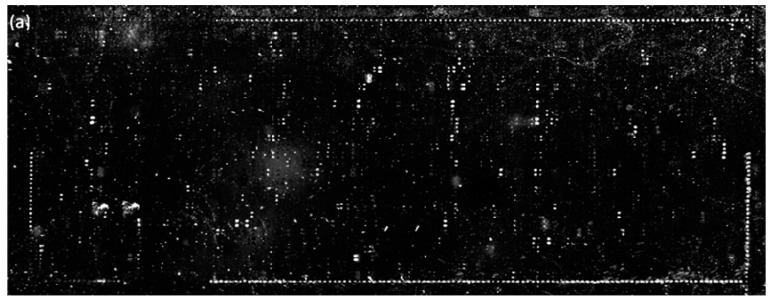

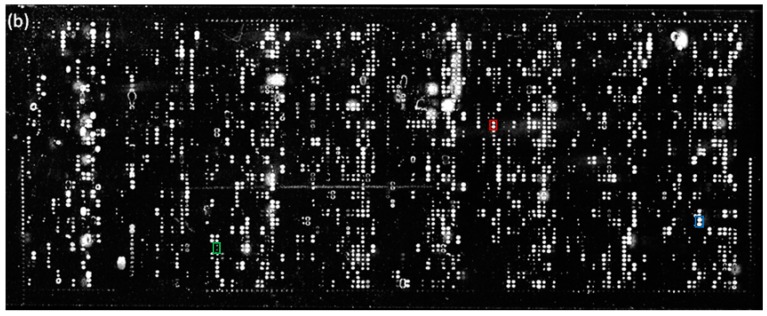
Combined auto-fluorescence/OI-RD image of a printed-and-washed compound microarray (**a**) on a hexyl-isocyanate functionalized slide and (**b**) on a phenyl-isocyanate functionalized slide.

SMMs of 3375 bioactive compounds are prepared on hexyl-isocyanate functionalized glass slides and on phenyl-isocyanate functionalized slides followed by thermal annealing at 45 °C for 24 h. From OI-RD images of printed microarrays before washing (Figure S2), 2982 compounds are successfully transferred (not necessarily immobilized via nucleophile-isocyanate reaction) to the surface. The remaining 393 compounds are not transferred to the surface, due to poor wetting properties of their printing solutions on the isocyanate-functionalized surface. After the slides are washed with 1× PBS and dried, we take auto-fluorescence images (Figure S3a) and OI-RD images (Figure S3b) of these slides to identify compounds that are successfully immobilized through the nucleophile-isocyanate reaction. In the auto-fluorescence image, the immobilized compounds that are auto-fluorescent appear as bright doublets; those that are immobilized and yet not auto-fluorescent appear as dark doublets if excess materials from neighboring auto-fluorescent compounds are washed over them during the washing step and the unprinted region becomes fluorescent. Those immobilized compounds that are not auto-fluorescent and have no excess auto-fluorescent materials washed over them can be identified as bright doublets in the OI-RD image if they are immobilized enough for OI-RD detection. For the purpose of counting immobilized compounds on a washed and dried slide, we combine the auto-fluorescent image and the properly scaled corresponding OI-RD image of the slide. Figure 8a shows the combined auto-fluorescence/OI-RD image of a printed compound microarray on a hexyl-isocyanate functionalized slide. By counting bright and dark doublets in the image, we find that 1746 compounds, out of 2982 printed, are immobilized with an overall immobilization percentage of 59%. In comparison, Figure 8b shows the combined auto-fluorescence/OI-RD image of a printed compound microarray on a phenyl-isocyanate functionalized slide. One can immediately see that there are many more bright and dark doublets. We identified 2165 compounds, out of 2982 printed, that are immobilized successfully with a markedly improved immobilization percentage of 73%. Considering that the combined image will miss those immobilized compounds (doublets) that (i) are not auto-fluorescent, (ii) have no excess auto-fluorescent materials washed over them, and (iii) are too small to be detected in our current OI-RD scanning microscope, the actual immobilization percentage is expected to be higher than 73%. In addition, three compounds with different nucleophilic functional groups (*i.e.*, amine, phenol, carboxylic acid) are highlighted in color rectangles in Figure 8b and listed in Table 1, indicating effective immobilization of compounds with nucleophilic groups on a phenyl-isocyanate functionalized surface. 

### 3.6. Screening Microarrays of~3000 Compounds for Protein Ligands—Further Confirmation of Improved Immobilization Efficiencies in Terms of Identified Ligands 

To examine whether the improvement in immobilization efficiency of a compound collection indeed leads to a corresponding increase in the number of protein ligand discovered on the SMM platform, we use streptavidin as a protein probe and incubate microarrays of 3375 compounds printed on hexyl- and phenyl-isocyanate functionalized slides in the solution of streptavidin at 154 nM for 1 h. Both microarrays are treated with thermal annealing at 45 °C for 24 h before the reaction. From changes in OI-RD image (Figure S4), 15 compounds on the hexyl-isocyanate slide reacted with streptavidin. On the phenyl-isocyanate slide, in addition to these 15 compounds, 7 additional compounds are found to react with streptavidin. Based on Figure 7 and Figure 8, this is expected as more compounds from the collection of 3375 compounds are immobilized on the phenyl-isocyanate slide. All 22 hit compounds (*i.e.*, ligands of streptavidin) are listed in Table 2, each having at least one of the following nucleophilic residues with different isocyanate reactivity: primary amine, aryl amine, secondary amine, phenol, and carboxylic acid. Among the seven additional hit compounds discovered on the phenyl-isocyanate slide, GW4064 only has a carboxylic acid for immobilization, confirming that the phenyl-isocyanate surface is indeed better for immobilization of small molecules with low isocyanate reactivity. 

## 4. Discussion

We investigated several surface chemistry parameters in fabrication of SMMs on isocyanate functionalized glass slides for optimal immobilization efficiency, spot morphology and reproducibility. 

We find that both the penultimate group next to the isocyanate reside and post-printing treatment have significant impacts on immobilization efficiencies of small molecules, particularly those having residues with inherently low reactivity with isocyanate. Both presumably facilitate covalent attachment of nucleophilic groups to the isocyanate residues on the functionalized surface. In particular, we find that a phenyl-isocyanate functionalized surface is more efficient for small molecule immobilization than a hexyl-isocyanate functionalized surface by almost a factor of two. We further find that thermal annealing of printed small molecule microarrays at 45 °C yields higher immobilization efficiency by almost another factor of two than the method of catalyzation in pyridine vapor at room temperature [25,27]. As a result, a combination of phenyl-isocyanate functionalized surface and annealing fabricated microarrays at 45 °C dramatically enhances immobilization efficiency, particularly for compounds with nucleophilic residues such as hydrazide, primary hydroxyl, and carboxylic acid. The overall improvement of immobilization efficiency and spot morphology are confirmed with SMMs of 3375 bioactive compounds printed on both phenyl-isocyanate and hexyl-isocyanate surfaces.

In addition we find that a (PEG)_n_ spacer with n = 6 is optimal in yielding high-quality SMM spot morphology while minimizing non-specific protein binding to the surface. Furthermore, we find that isocyanate functionalized surfaces degrade after storage in −20 °C freezer for a period of time. Ambient moisture reacts with surface isocyanate residue slowly and causes the surface density of the isocyanate residues to diminish in time. It is therefore important to prepare isocyanate functionalized glass slides and use them immediately. On the other hand, water present in the printing solution has little effect on the immobilization efficiency of small molecules, presumably due to rapid evaporation of deposited solution droplets. Insensitivity of immobilization efficiency to water in a printing solution is a pre-requisite for fabrication of SMMs, as ambient moisture is easily absorbed into DMSO during long printing runs.

In conclusion, we find that in combination with post-printing thermal annealing treatment at 45 °C, a phenyl-isocyanate functionalized glass surface with (PEG)_6_ as the spacer between the phenyl-isocyanate residues and the solid surface is optimal for fabrication of SMMs. The high immobilization percentage (over 73%) and spot morphology of SMMs on such surfaces should improve the quality and the range of SMM applications in high-throughput drug lead discovery.

## Figures and Tables

**Table 1:** Structures of three light spots in Figure 8b highlighted by color rectangles.

Compound Names	Compound Structures	Rectangular Color
NVP-AEW541	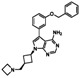	red
WY-14643	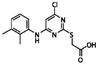	green
Arbidol HCl	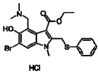	blue

**Table 2:** Hit compounds and surface coverage of SAVD on both a phenyl-isocyanate surface and hexyl-isocyanate surface.

Compound Names	Compound Structures	SAVD Coverage on Phenyl-Isocyanate Surface	SAVD Coverage on Hexyl-Isocyanate Surface
BMS 777607	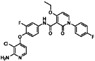	0.85	0.54
JNJ-7706621	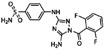	0.85	0.28
Biotin-NH2	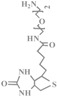	0.73	0.61
Azelnidipine	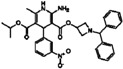	0.64	0.28
Amfenac sodium monohydrate	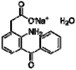	0.64	0.64
Dryocrassin	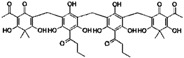	0.61	0.45
Magnolol	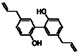	0.58	0.39
LDN193189	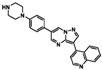	0.52	0.36
Manidipine	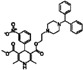	0.51	0.45
Tyrphostin AG 879	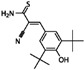	0.48	0.48
Honokiol	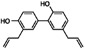	0.45	0.36
NVP-BSK805	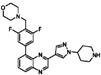	0.39	0.33
PF-04929113	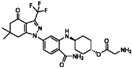	0.33	0.27
Flupirtine maleate	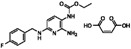	0.30	0.27
Linifanib (ABT-869)	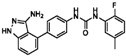	0.27	0.18
Sciadopitysin	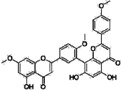	0.36	0
ABT-263 (Navitoclax)	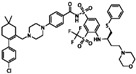	0.33	0
GW4064	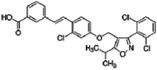	0.29	0
MLN8054	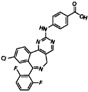	0.27	0
Flunarizine dihydrochlor	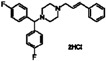	0.26	0
Nomilin	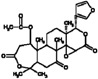	0.25	0
Liothyronine sodium	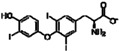	0.18	0

## Supplementary Materials

Supplementary materials can be accessed at: http://www.mdpi.com/1424-8220/16/3/378/s1.

## References

[1] Sun H.Y., Chen G.Y.J., Yao S.Q. (2013). Recent advances in microarray technologies for proteomics. Chem. Biol..

[2] He X.Z.G., Gerona-Navarro G., Jaffrey S.R. (2005). Ligand discovery using small molecule microarrays. J. Pharmacol. Exp. Ther..

[3] Hong J.A., Neel D.V., Wassaf D., Caballero F., Koehler A.N. (2014). Recent discoveries and applications involving small-molecule microarrays. Curr. Opin. Chem. Biol..

[4] Wang J., Uttamehandani M., Sun H.Y., Yao S.Q. (2006). Small molecule microarrays: Applications using specially tagged chemical libraries. Qsar. Comb. Sci..

[5] Casalena D., Wassaf D., Koehler A., Drewes G., Bantscheff M. (2012). Ligand discovery using small-molecule microarrays. Chemical proteomics.

[6] Zhang B., Jarrell J.A., Price J.V., Tabakman S.M., Li Y.G., Gong M., Hong G.S., Feng J., Utz P.J., Dai H.J. (2013). An integrated peptide-antigen microarray on plasmonic gold films for sensitive human antibody profiling. PLoS One.

[7] Lesaicherre M.L., Uttamchandani M., Chen G.Y.J., Yao S.Q. (2002). Developing site-specific immobilization strategies of peptides in a microarray. Bioorg. Med. Chem. Lett..

[8] Gao L.Q., Uttamchandani M., Yao S.Q. (2012). Comparative proteomic profiling of mammalian cell lysates using phosphopeptide microarrays. Chem. Commun..

[9] Uttamchandani M., Lee W.L., Wang J., Yao S.Q. (2007). Quantitative inhibitor fingerprinting of metalloproteases using small molecule microarrays. J. Am. Chem. Soc..

[10] Sun H.Y., Lu C.H.S., Shi H., Gao L.Q., Yao S.Q. (2008). Peptide microarrays for high-throughput studies of ser/thr phosphatases. Nat. Protoc..

[11] Gao L., Lee S.S., Chen J., Sun H., Zhao Y., Chai Z., Hu Y. (2016). High-throughput screening of substrate specificity for protein tyrosine phosphatases (ptps) on phosphopeptide microarrays. Methods Mol. Biol..

[12] Gao L., Sun H., Uttamchandani M., Yao S.Q. (2013). Phosphopeptide microarrays for comparative proteomic profiling of cellular lysates. Methods Mol. Biol..

[13] Gao L.Q., Sun H.Y., Yao S.Q. (2010). Activity-based high-throughput determination of ptps substrate specificity using a phosphopeptide microarray. Biopolymers.

[14] MacBeath G., Koehler A.N., Schreiber S.L. (1999). Printing small molecules as microarrays and detecting protein-ligand interactions en masse. J. Am. Chem. Soc..

[15] Hergenrother P.J., Depew K.M., Schreiber S.L. (2000). Small-molecule microarrays: Covalent attachment and screening of alcohol-containing small molecules on glass slides. J. Am. Chem. Soc..

[16] Barnes-Seeman D., Park S.B., Koehler A.N., Schreiber S.L. (2003). Expanding the functional group compatibility of small-molecule microarrays: Discovery of novel calmodulin ligands. Angew. Chem. Int.Edit..

[17] Lee M.R., Shin I. (2005). Fabrication of chemical microarrays by efficient immobilization of hydrazide-linked substances on epoxide-coated glass surfaces. Angew. Chem. Int.Edit..

[18] Fazio F., Bryan M.C., Blixt O., Paulson J.C., Wong C.H. (2002). Synthesis of sugar arrays in microtiter plate. J. Am. Chem. Soc..

[19] Kohn M., Wacker R., Peters C., Schroder H., Soulere L., Breinbauer R., Niemeyer C.M., Waldmann H. (2003). Staudinger ligation: A new immobilization strategy for the preparation of small-molecule arrays. Angew. Chem. Int. Edit..

[20] Ko K.S., Jaipuri F.A., Pohl N.L. (2005). Fluorous-based carbohydrate microarrays. J. Am. Chem. Soc..

[21] Urbina H.D., Debaene F., Jost B., Bole-Feysot C., Mason D.E., Kuzmic P., Harris J.L., Winssinger N. (2006). Self-assembled small-molecule microarrays for protease screening and profiling. ChemBioChem.

[22] Kanoh N., Kumashiro S., Simizu S., Kondoh Y., Hatakeyama S., Tashiro H., Osada H. (2003). Immobilization of natural products on glass slides by using a photoaffinity reaction and the detection of protein-small-molecule interactions. Angew. Chem. Int.Edit..

[23] Bradner J.E., McPherson O.M., Mazitschek R., Barnes-Seeman D., Shen J.P., Dhaliwal J., Stevenson K.E., Duffner J.L., Park S.B., Neuberg D.S. (2006). A robust small-molecule microarray platform for screening cell lysates. Chem. Biol..

[24] Kurosu M., Mowers W.A. (2006). Small-molecule microarrays: Development of novel linkers and an efficient detection method for bound proteins. Bioorg. Med. Chem. Lett..

[25] Bradner J.E., McPherson O.M., Koehler A.N. (2006). A method for the covalent capture and screening of diverse small molecules in a microarray format. Nat. Protoc..

[26] Lee H.Y., Park S.B. (2011). Surface modification for small-molecule microarrays and its application to the discovery of a tyrosinase inhibitor. Mol. Biosys..

[27] Landry J.P., Fei Y.Y., Zhu X.D., Ke Y.H., Yu G.L., Lee P. (2013). Discovering small molecule ligands of vascular endothelial growth factor that block VEGF-KDR binding using label-free microarray-based assays. Assay Drug Dev. Technol..

[28] Chen J., Armstrong A.H., Koehler A.N., Hecht M.H. (2010). Small molecule microarrays enable the discovery of compounds that bind the alzheimer's a beta peptide and reduce its cytotoxicity. J. Am. Chem. Soc..

[29] Lopez-Paz J.L., Gonzalez-Martinez M.A., Escorihuela J., Banuls M.J., Puchades R., Maquieira A. (2014). Direct and label-free monitoring oligonucleotide immobilization, non-specific binding and DNA biorecognition. Sens. Actuator B-Chem..

[30] Vigano M., Levi M., Turri S., Chiari M., Damin F. (2007). New copolymers of n,n-dimethylacrylamide with blocked isocyancates for oligonucleotide immobilization in DNA microarray technology. Polymer.

[31] Xu Y.L., Xu H.J., Jiang X.S., Yin J. (2014). Versatile functionalization of the micropatterned hydrogel of hyperbranched poly(ether amine) based on "thiol-yne" chemistry. Adv. Funct. Mater..

[32] Vigano M., Suriano R., Levi M., Turri S., Chiari M., Damin F. (2007). Glass silanization with blocked-isocyanate for the fabrication of DNA microarrays. Surf. Sci..

[33] Fei Y.Y., Schmidt A., Bylund G., Johansson D.X., Henriksson S., Lebrilla C., Solnick J.V., Boren T., Zhu X.D. (2011). Use of real-time, label-free analysis in revealing low-affinity binding to blood group antigens by helicobacter pylori. Anal. Chem..

[34] Fei Y.Y., Landry J.P., Sun Y.S., Zhu X.D., Luo J.T., Wang X.B., Lam K.S. (2008). A novel high-throughput scanning microscope for label-free detection of protein and small-molecule chemical microarrays. Rev. Sci. Instrum..

[35] Landry J.P., Fei Y., Zhu X. (2012). Simultaneous measurement of 10,000 protein-ligand affinity constants using microarray-based kinetic constant assays. Assay Drug Dev. Technol..

[36] Fei Y., Sun Y., Li Y., Yu H., Lau K., Landry J., Luo Z., Baumgarth N., Chen X., Zhu X. (2015). Characterization of receptor binding profiles of influenza a viruses using an ellipsometry-based label-free glycan microarray assay platform. Biomolecules.

[37] Liu S., Zhu J.H., He L.P., Dai J., Lu H.B., Wu L., Jin K.J., Yang G.Z., Zhu H. (2014). Label-free, real-time detection of the dynamic processes of protein degradation using oblique-incidence reflectivity difference method. Appl. Phys. Lett..

[38] Sun Y.S., Landry J.P., Fei Y.Y., Zhu X.D., Luo J.T., Wang X.B., Lam K.S. (2009). Macromolecular scaffolds for immobilizing small molecule microarrays in label-free detection of protein-ligand interactions on solid support. Anal. Chem..

[39] Golander C.G., Kiss E. (1988). Protein adsorption on functionalized and esca-characterized polymer-films studied by ellipsometry. J. Colloid Interface Sci..

[40] Sun Y.S., Landry J.P., Fei Y.Y., Zhu X.D. (2008). Effect of fluorescently labeling protein probes on kinetics of protein-ligand reactions. Langmuir.

[41] Uchida K., Otsuka H., Kaneko M., Kataoka K., Nagasaki Y. (2005). A reactive poly(ethylene glycol) layer to achieve specific surface plasmon resonance sensing with a high s/n ratio: The substantial role of a short underbrushed peg layer in minimizing nonspecific adsorption. Anal. Chem..

[42] Torres-Lugo M., Garcia M., Record R., Peppas N.A. (2002). Physicochemical behavior and cytotoxic effects of p(methacrylic acid-g-ethylene glycol) nanospheres for oral delivery of proteins. J. Controlled Release.

