# Supplementary material for: Developing an Efficient and General Strategy for Immobilization of Small Molecules onto Microarrays Using Isocyanate Chemistry
Source: Sensors (Basel). 2016 Mar 16;16(3):378. doi: 10.3390/s16030378 (PMC4813953; doi:10.3390/s16030378)
Supplement: Supplementary file 1 [file sensors-16-00378-s001.pdf]

Supplementary Materials: Developing an Efficient and General Strategy for Immobilization of Small Molecules onto Microarrays Using Isocyanate Chemistry

Chenggang Zhu, Xiangdong Zhu, James P. Landry, Zhaomeng Cui, Quanfu Li, Yongjun Dang, Lan Mi, Fengyun Zheng and Yiyan Fei

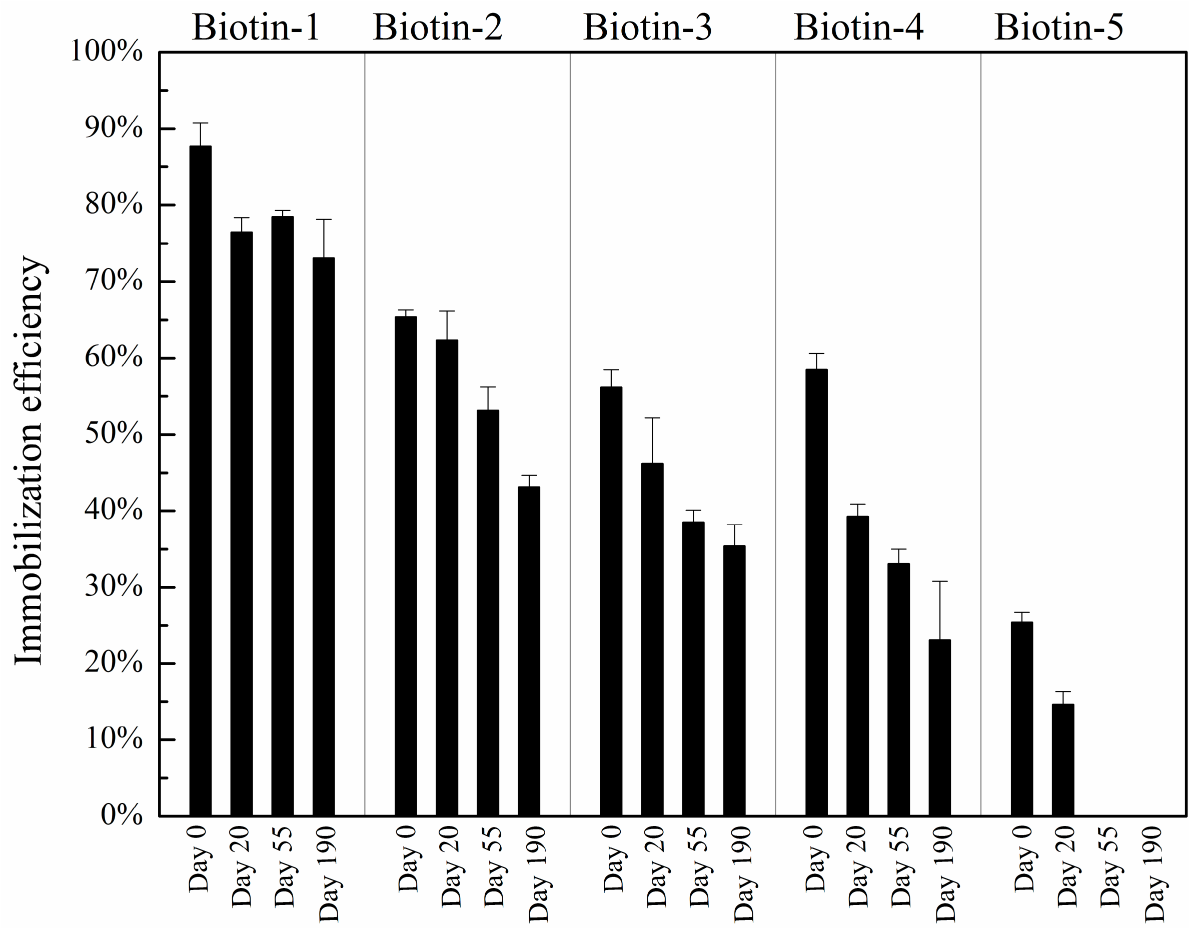

Figure S1. Immobilization efficiencies of five biotinylated compounds on phenyl-isocyanate functionalized slides as functions of storage time.

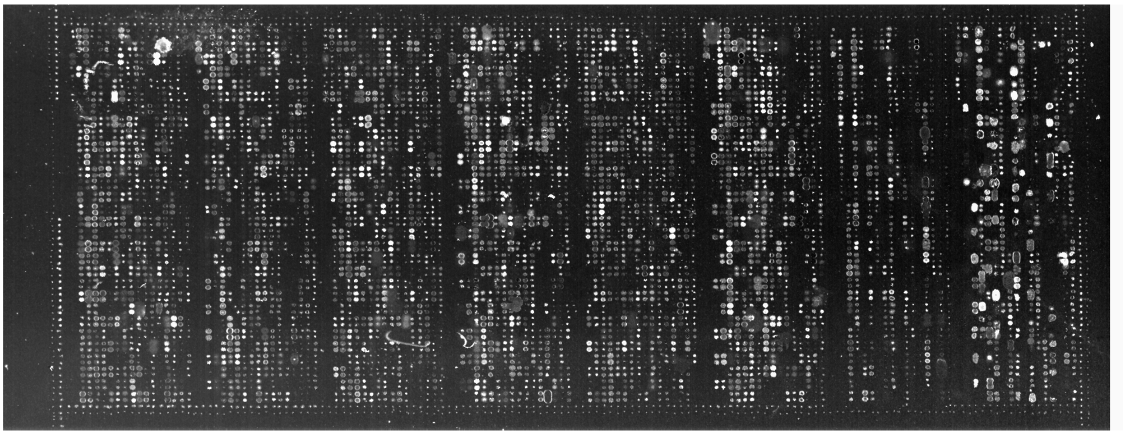

Figure S2. OI-RD image of a small molecule microarray printed from a collection of 3375 bioactive compounds on a phenyl-isocyanate functionalized slide. The image was acquired under dry condition before washing. Solutions of 2982 compounds were successfully transferred to the slide from the microplate during printing. Solutions of the remaining 393 compounds were not transferred due to poor wetting properties on the isocyanate surface.

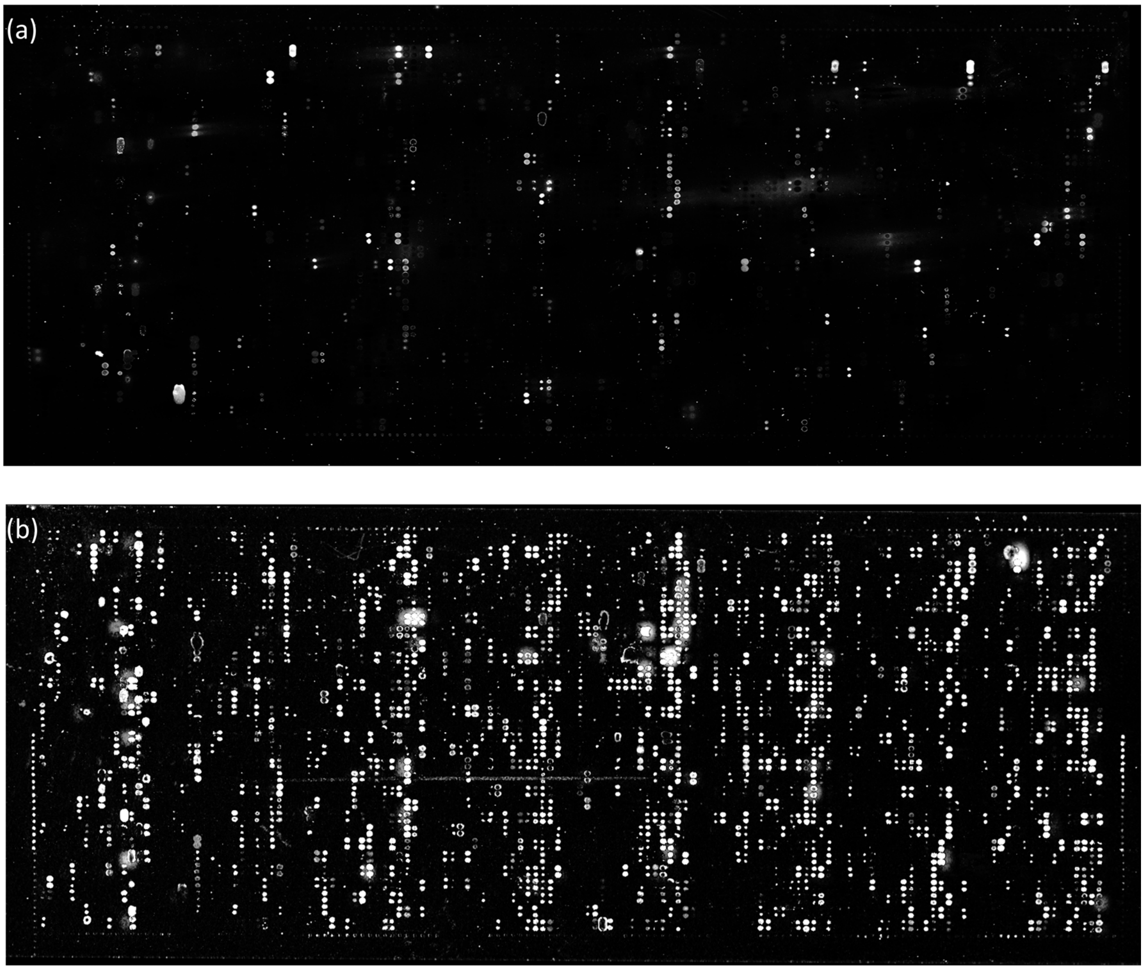

Figure S3. (**a**) Auto-fluorescence image and (**b**) OI-RD image of SMM after washing.

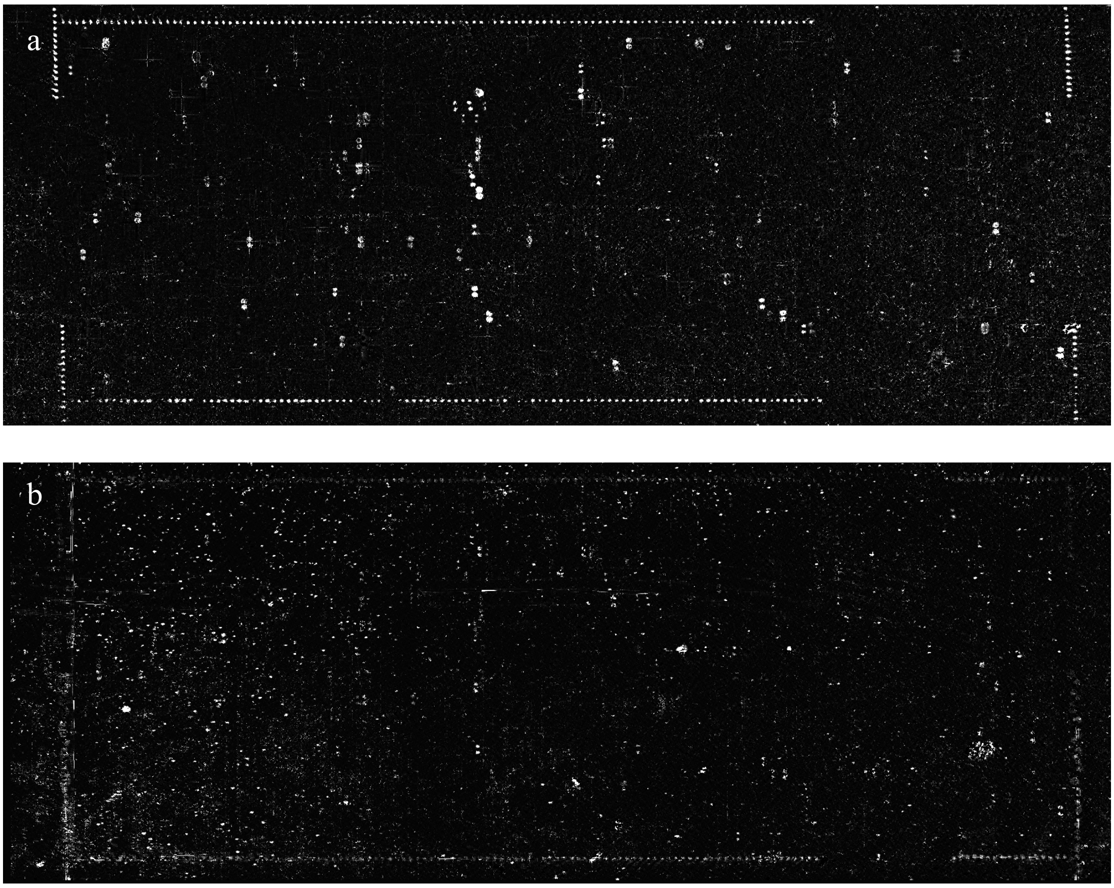

Figure S4. Changes after reaction with streptavidin in OI-RD image of small molecule microarrays with 3375 compounds printed on (**a**) phenyl-isocyanate functionalized slide and (**b**) hexyl-isocyanate functionalized slide, respectively. 22 compounds were identified to have reacted with streptavidin on phenyl-isocyanate surface, while only 15 of them were identified on hexyl-isocyanate surface.
